# Resting-state functional connectivity patterns are associated with worst pain duration in community-dwelling older adults

**DOI:** 10.1097/PR9.0000000000000978

**Published:** 2021-12-08

**Authors:** Pedro A. Valdes-Hernandez, Soamy Montesino-Goicolea, Lorraine Hoyos, Eric C. Porges, Zhiguang Huo, Natalie C. Ebner, Adam J. Woods, Ronald Cohen, Joseph L. Riley, Roger B. Fillingim, Yenisel Cruz-Almeida

**Affiliations:** a Department of Community Dentistry and Behavioral Science, University of Florida, USA; b Pain Research and Intervention Center of Excellence, Department of Community Dentistry and Behavioral Science, College of Dentistry, University of Florida, Gainesville, Florida, USA; c Center for Cognitive Aging and Memory, McKnight Brain Institute, College of Medicine, University of Florida, Gainesville, Florida, USA; d University of Central Florida, Department of Clinical Sciences, Orlando, Florida, USA; e Department of Clinical and Health Psychology, College of Public Health and Health Professions, University of Florida, Gainesville, Florida, USA; f Department of Biostatistics, College of Public Health and Health Professions and College of Medicine, University of Florida, Gainesville, Florida, USA; g Department of Psychology, College of Liberal Arts and Sciences, University of Florida, Gainesville, Florida, USA; h Department of Neuroscience, College of Medicine, University of Florida, Gainesville, Florida, USA

**Keywords:** Musculoskeletal pain, Pain duration, Resting-state functional connectivity, Older adults, Visuospatial network, Salience-DMN connectivity

## Abstract

**Introduction:**

An individual's chronic pain history is associated with brain morphometric alterations; but little is known about the association between pain history and brain function.

**Objectives:**

This cross-sectional study aimed at determining how worst musculoskeletal pain intensity (WPINT) moderated the association between worst musculoskeletal pain duration (WPDUR) and brain resting-state magnetic resonance imaging functional connectivity (RSFC) in community-dwelling older adults (60–94 years, 75% females, 97% right-handed).

**Methods:**

Resting-state magnetic resonance imaging functional connectivity between region of interests was linearly regressed on WPDUR and WPINT. Predictions were compared with a control group's average RSFC (61–85 years, 47% females, 95% right-handed).

**Results:**

Three significant patterns emerged: (1) the positive association between WPDUR and RSFC between the medial prefrontal cortex, in the anterior salience network (SN), and bilateral lateral Brodmann area 6, in the visuospatial network (VSN), in participants with more severe chronic pain, resulting in abnormally lower RSFC for shorter WPDUR; (2) the negative association between WPDUR and RSFC between right VSN occipitotemporal cortex (lateral BA37 and visual V5) and bilateral VSN lateral Brodmann area 6, independently of WPINT, resulting in abnormally higher and lower RSFC for shorter and longer WPDUR, respectively; and (3) the positive association between WPDUR and the left hemisphere's salience network-default mode network connectivity (between the hippocampus and both dorsal insula and ventral or opercular BA44), independently of WPINT, resulting in abnormally higher RSFC for longer WPDUR.

**Conclusion:**

Musculoskeletal effects on brain functional networks of general healthy individuals could accumulate until being observable at older ages. Results invite to examinations of these effects' impact on function and memory.

## Abstract 2

Supplemental Digital Content is Available in the Text. Musculoskeletal pain duration is associated with brain resting-state functional magnetic resonance imaging connectivity within the visuospatial network and between salience-default networks in older adults.

## 1. Introduction

The neurobiological mechanisms that facilitate the high prevalence of chronic pain among community-dwelling older adults are currently unclear, hindering the development of effective strategies to attenuate or alleviate the ensuing pain-related mobility limitations and disabilities common in this group.^18,61,72^ The multidimensional pain experience is sculpted by dynamic interactions in the brain. That is why case–control studies using magnetic resonance imaging (MRI) have already identified alterations associated with chronic pain in brain morphometry^14,57,75^ and function.^33,56,68^ Additional MRI studies examining how such alterations gradually accumulate over time until old age are also needed.

Chronic pain–related differences in the brain could be preexisting traits of pain vulnerability or plastic adaptation caused by transitional chronification processes and later maintenance phases. A number of biobehavioral factors may predispose individuals to establish aberrant brain patterns across the lifespan by multiple neurobiological mechanisms. For example, prolonged reductions in mesolimbic thresholds controlling the conversion from nociception to conscious pain perception^11^ may expose the brain to persistent painful inputs and associated aversive states. These may be amplified through synaptic plasticity and learning mechanisms, eventually causing permanent maladaptive structural reorganization, synaptic dysregulation, and distorted functional connectivity (FC).^11,17,33,40^ This reverberating process is potentially maintained beyond the transitional period of pain chronification and may direct the brain toward a pain-addictive state that possibly activates unfolding mechanisms of learning, resilience, and coping^48^ that consolidate over the decades.^65^ Irrespective of the possible underlying mechanisms, the changes that occur in response to pain chronification, persistence, and long-term management likely accumulate and correlate with the number of years individuals experience chronic pain; whereas those related to preexisting traits of pain vulnerability likely remain unchanged.^3^ Thus, examination of the correlation between brain structure or function and variables characterizing the history or burden of pain may help determine which brain networks are involved in the mechanisms underlying long-term pain experience, as well as resistance to interventions.

Several studies have already examined correlations between brain morphology and pain duration in younger and middle-age individuals,^6,55,64,71,82^ with fewer extending into older adults.^3,14,19^ Morphometric alterations are expected to underlie aberrant patterns of resting-state MRI FC (RSFC),^21^ reflecting the history of perceptual learning and memory,^60^ and the experience-dependent brain plasticity^39^ of an individual. However, there is currently no report of a significant pain duration FC association in community-dwelling older adults with musculoskeletal pain, despite their generally longer pain histories (eg, >40 years^3,14,19^)—such paucity may owe in part to a scant literature about the effects of chronic pain on RSFC in older adults (to the best of our knowledge, only 2 studies to date^19,35^). In general, we find only 3 studies reporting significant correlations between chronic pain duration and RSFC in middle-age adults. In a migraine study, a significant correlation was reported between disease duration and default mode-salience networks decoupling.^46^ Conversely, another study reported a significant positive correlation between pain duration and default mode network (DMN)-left insula RSFC in fibromyalgia patients with no clinical pain during functional MRI (fMRI) acquisition.^23^ Another fibromyalgia study found that pain duration was inversely associated with the RSFC within the DMN.^38^

The aim of this investigation was to evaluate the association between self-reported pain history and brain RSFC in a cohort of community-dwelling older adults with musculoskeletal pain. Because the cumulative effects of chronic pain over time are often modified by a number of factors including its intensity,^34^ pain duration alone might not be enough to reflect the negative impact of chronic pain burden on an individual's life.^53^ We expect the accumulated burden associated with different chronic pain severity levels to be different over the same period of years. Thus, we hypothesized that pain duration correlates with RSFC (either within or between) of the default mode, salience, executive, attention, sensorimotor, and subcortical networks, known to be affected by chronic pain^2,28,33,41,56,59^ and that this correlation is moderated by the severity of pain. Of note, testing this hypothesis is equivalent to testing how the effects of the severity of pain are moderated by its duration.

## 2. Materials and methods

### 2.1. Participants

Community-dwelling, native English speakers older adults (≥60 years) were recruited through posted fliers, ads, and word of mouth referrals between January 2016 and January 2018 as part of an ongoing project at the University of Florida (UF) (Neuro-modulatory Examination of Pain and Mobility Across the Lifespan [NEPAL]). The study was approved by the UF Institutional Review Board. Potential participants were screened over the phone and again in person. The exclusionary criteria included Alzheimer Disease, Parkinson Disease, or other conditions directly affecting the brain; serious psychiatric conditions (eg, schizophrenia, major depression, or bipolar disorder); uncontrolled hypertension (blood pressure ≥150/95 mm Hg), heart failure, or history of acute myocardial infarction; systemic rheumatic disorders (ie, rheumatoid arthritis, systemic lupus erythematosus, or fibromyalgia); chronic opioid use; MRI contraindications; excessive anxiety regarding protocol procedures; hospitalization within the preceding year for psychiatric illness; HIV or AIDS; and cognitive impairment (Modified Mini-Mental State [3 MS] Examination score < 77^77^); or inability to provide consent for study participation. During a baseline visit, on verbal and written informed consent, participants completed general health and demographic information questionnaires.

The main analysis of this study, that is the determination of the accumulated effects of pain history on RSFC, is based on a subsample of the NEPAL study of older participants that self-reported pain for the past 3 months on most days (n_p_ = 40, pain group). An additional subset of older participants of the NEPAL study that did not report pain (n_c_ = 21, control group) was used in a follow-up analysis to determine how much the detected pain history effects deviated RSFC from “normal” values.

### 2.2. Main pain history variables

Following the same procedures of previous articles from our group,^29,31^ during the baseline visit, participants completed a standardized pain history interview regarding the presence of pain across several body regions (ie, head/face, neck, shoulders, arms, hands, chest, stomach, upper and lower back, leg, knees, and feet) using a validated body manikin.^30,63^ This is important as older adults often present with more than one type of pain; thus, each pain should be evaluated separately to the extent possible. Participants were asked to rank the location of their worst, second worst, and third worst pain and asked about its frequency during the past week and how hard it was to deal with their worst pain. They were also asked to provide the number of years the participants reported having each pain problem (ie, pain duration) and its intensity on average, as assessed using an 11-point Numerical Rating Scale (0 = no pain and 10 = worst imaginable pain).

The main independent variables of the analysis on the pain group were the duration of the worst pain (WPDUR) and its intensity (WPINT). If only one pain was present, we considered that pain the worst pain. We modeled the effect of WPINT on RSFC using linear relationships. To model how pain duration could affect RSFC, we followed previous approaches in the neuroimaging literature:

(1) Logarithmic approach: testing the association between RSFC and logWPDUR = log_10_(WPDUR). This approach is consistent with previous studies.^3,14^
(2) Linear approach: testing the association between RSFC and WPDUR. This approach was used in Refs. 6,^19^,^23^,^46^,^55^,^64^,^71^, and 82.
(3) Dichotomous approach: testing the difference in RSFC among the levels of the dichotomous variable WPDUR5, with levels WPDUR ≤ 5 years (n = 23) and WPDUR > 5 years (n = 17). This stratification was proposed in Ref. 14.

In addition, note that each approach allows to test different possible ways by which pain burden gradually affects RSFC. For example, although longer pain durations are expected to have stronger accumulated effects than shorter ones for a given pain intensity, the logarithmic approach allows to test if the rate of these accumulations is different for different pain onsets. Under this approach, the effects of pains with onsets at later ages (ie, shorter pain durations) could have higher rates of impact on the brain than those that onset earlier in life (ie, longer pain durations), provided that we account or control for the effects of age at the time of the MRI scan (ie, as if all ages were the same at the time of the study). This is because, in a logarithmic fit of RSFC against pain duration, shorter pain durations correspond to steeper slopes. This will be better appreciated in the results when changing the RSFC vs logWPDUR plot to a RSFC vs WPDUR one.

### 2.3. Magnetic resonance imaging acquisition and preprocessing

Brain images were acquired at the UF's McKnight Brain Institute with a 3T Philips Achieva MR Scanner (Philips Medical Systems, Best, The Netherlands) using a 32-channel head coil. Before starting the MRI sequences, but after lying down on the MRI table, participants were asked to verbally rate their current level of pain using a Numerical Rating Scale from 0 to 100, where 0 = no pain and 100 = worst bearable pain. Resting-state gradient echo-planar imaging data (fMRIs) were acquired with 36 Philips-interleaved slices, TR = 2 seconds, TE = 30 milliseconds, field-of-view (FOV) = 224 × 224 × 133 mm, 64 × 64 × 36 matrix, flip angle = 90°, in plane resolution = 3.5 × 3.5 mm, slice thickness = 3.5 mm, 0 mm skip, and SENSE factor = 2 in the AP direction. The run lasted 10 minutes, and 300 time points were acquired. Whole-brain high-resolution 3-dimensional T1-weighted anatomical images were also acquired using an MP-RAGE sequence with sagittal plane, FOV = 240 × 240 × 170 mm, 1 × 1 × 1 mm isotropic voxels, TR = 7.1 milliseconds, TE = 3.2 milliseconds, and flip angle = 8°. During the acquisition, participants stared at a cross in the middle of a screen.

We preprocessed fMRIs using standard SPM12 (www.fil.ion.ucl.ac.uk/spm) pipelines for slice timing and motion or unwarp correction. We used SPM12's unified segmentation^9^ to segment time averaged fMRIs into gray matter (GM) or white matter (WM) and cerebrospinal fluid (CSF) and to spatially normalize them to the Montreal Neurological Institute space. Given the remaining large morphometric variability in the sample, we refined normalization using SPM12's Diffeomorphic Anatomical Registration Through Exponentiated Lie algebra (DARTEL) with default parameters^8^ to generate sample-specific template segmentations in the Montreal Neurological Institute space with a final resolution of 3 × 3 × 3 mm. Because DARTEL delivers large deformations, we used the *pushforward* warping method to preserve the data from the native fMRIs.^10^ We applied the same segmentation and DARTEL procedures independently to the T1-weighted images. GM, WM, and CSF masks were eroded with a binarization threshold of 0.3, 0.5, and 0.5, respectively, one erosion step and one erosion neighbor.

### 2.4. Outcomes: resting-state magnetic resonance imaging functional connectivity

For each participant, we calculated the average within 76 region of interests (ROIs) of the preprocessed fMRIs, excluding voxels outside the individual's gray matter mask, using CONN ver. 19a.^80^ ROIs were defined from a predefined atlas of functional ROIs defined across 10 large-scale resting-state brain networks, previously obtained with group independent component analysis, covering most cortical and subcortical gray mater.^74^ By definition, these functional ROIs can cover several different (contiguous) anatomical cortical, subcortical, and cerebellar structures. The functional networks are the dorsal DMN, ventral DMN, precuneus network, sensorimotor network, visuospatial network (VSN), anterior salience network, posterior SN, right and left executive central networks, and basal network. We clarify that some ROIs' roles are reportedly not restricted to what their network names suggest (eg, the premotor ROIs in the VSN).

We temporally filtered the ROI time series using a band-pass filter between 0.008 and 0.09 Hz and denoised both the unfiltered and filtered time series using the general linear model. Noise regressors were the 6 motion parameters and their temporal derivatives, *scrubbing* (acquisitions with frame-wise displacement above 0.9 mm or global BOLD signal changes above 5 standard deviations were flagged as potential outliers), the first 5 spatial principal components of the spatially nonsmoothed preprocessed fMRIs within white matter and CSF (*aCompCor* method^15^), and a 10-minute duration boxcar function (*rest* regressor) convolved with the canonical hemodynamic response function, as well as its first and second temporal derivatives. We converted the denoised fMRIs and ROI time series to percent signals and centered them to have zero mean. ROI-to-ROI (R2R) RSFC was defined as the zero-lagged bivariate weighted temporal correlation between a pair (or connection) of ROI time series, Fisher transformed to have normal distribution.

Using CONN's visualizer, quality control (QC) of the processing was based on (1) visual comparison, before and after denoising, of the histograms of temporal correlation between random 1000 brain voxels of the preprocessed fMRIs, as well as on (2) visual inspection of the carpet plots of the BOLD signals in all voxels.^69^ We also evaluated departures from the null distribution, obtained with permutations, of the correlation (1) between the RSFC in a random 1000-node network and brain displacements and (2) between the former and global signal changes.^25^ We calculated a set of second-level QC variables based on the variability of the signal (BOLD standard deviation and mean and maximum BOLD change), the motion parameters (mean and maximum change in position after time-point outlier removal, ie, scrubbing), and the number of outlier scans (outliers, ie, nonzero values in the scrubbing regressors). We then inspected the violin plots of the mean signal change, mean motion, and invalid scans to find participants outside 3 times the interquartile range of their sample distribution. Three subjects in the pain group were classified as potential outliers because of a high number of outlier scans: they had a 29.3%, 48.6%, and 49.7% of invalid scans, whereas the rest were below 10%. The worst pain durations of these potential outliers were 0.25, 5, and 10 years, and their worst pain intensity were 7, 5, and 8, respectively. Because the standard practice is to consider fMRI scans with less than 50% of outlier time points (also known as “censored frames”) to contain valid and useful information, we did not remove these participants from our analyses.^52^ Nonetheless, as described below, we performed a post hoc analysis to evaluate the possible effect of the amount of outlier scans, as well as other QC variables, in our results.

### 2.5. Statistical analysis

For each of the 2850 possible connections, we fitted group-level linear models using the RSFC of the participants in the pain group as the dependent variable. Under each of the above-mentioned approaches to relate RSFC to WPDUR, ie, logarithmic, linear, and dichotomous comparison, we fitted the linear model, RSFC∼WPINT*X + Covariates (in Wilkinson notation), where the independent variable X is either logWPDUR, WPDUR, or WPDUR5. The notation WPINT*X entails an interaction WPINT:X term that quantifies how WPINT moderates the effect of X on RSFC.^1^ To avoid unnecessary overfitting or loss of statistical power, we adopted a stepwise backward elimination regression approach,^44^ going from second-order to first-order terms but always keeping the X term (ie, WPINT:X followed by WPINT). At each elimination step, a term was removed if its corresponding F-statistic (T-contrast) had a *P* > 0.05 corrected for multiple comparisons using false discovery rate (FDR) across all connections. Covariates were sex, age, current use of pain medications (coded as a binary variable [no, yes]), the Montreal Cognitive Assessment (MoCA) score,^54^ and the clinical pain intensity at the scanner, given its association with DMN-insula RSFC in individuals with fibromyalgia.^23^ The rationale for the inclusion of these covariates (and exclusion of others) can be found in Supplemental Material S1 (available at http://links.lww.com/PR9/A141).

To evaluate how effects of the pain history variables deviate RSFC from normal values, we statistically compared the adjusted RSFC values predicted by the models above (fitted in the pain group) with the estimated adjusted average of RSFC in the control group (see Supplemental Material S2 for mathematical details, available at http://links.lww.com/PR9/A141). The latter was the intercept term of RSFC∼Covariates, where covariates were sex, age, and MoCA score. Statistical significance of the comparison was set to the FDR q-value resulting from the corresponding regression in the pain group.

## 3. Results

Participants in the pain group were between 61 and 94 years old, mostly females (75%); and mostly right-handed (97%). They reported chronic musculoskeletal pain locations in the upper or lower back but not in the knees (25%), in the knees but not in the back (36%), in both back and knees (22%), and in other locations (neck, shoulder, arm, hands, thighs, or legs) but not in the back or the knees (17%). Participants had a relatively low clinical pain during the fMRI acquisition. Twenty-nine had mild clinical pain in the scanner, with 6 of them rating an intensity of less than 10/100 and 17 having no pain at all; and only 3 reported moderate pain between 35 and 45 (25/50/75 quartiles of 0/2/35 of 100).

The self-reported worst pain duration (WPDUR), had a mean, standard deviation, and range of 11.3, 14.7, and 0.25 to 56 years, as well as 25/50/75 quartiles of 1/5/14.25 years. In addition, WPDUR was relatively representative of the entire experience of pain because it strongly correlated (*r* = 0.74, *P* = 5.5 × 10^−8^) with the longest pain duration, ie, the maximum among the 3 worst pains. The worst pain intensity on average (WPINT) ranged from mild to severe (1–9 out of 10), with mean and standard deviation of 4.8 and 2.0, and 25/50/75 quartiles of 3/4.5/6.5.

Table 1 shows the summary statistics of the main pain history variables, the covariates, and other sample-descriptive measures quantifying possible pain-related comorbidities or disabilities (ie, measures of mood, sleep, and both physical and cognitive function; see the Supplemental Material S3 for details, available at http://links.lww.com/PR9/A141). The table also shows that pairwise correlation of all variables with the main pain history variables did not survive correction for multiple comparisons across the tests in the table (*P* > 0.05; FDR corrected).

**Table 1:** Statistical relation between possible covariates and pain history variables.

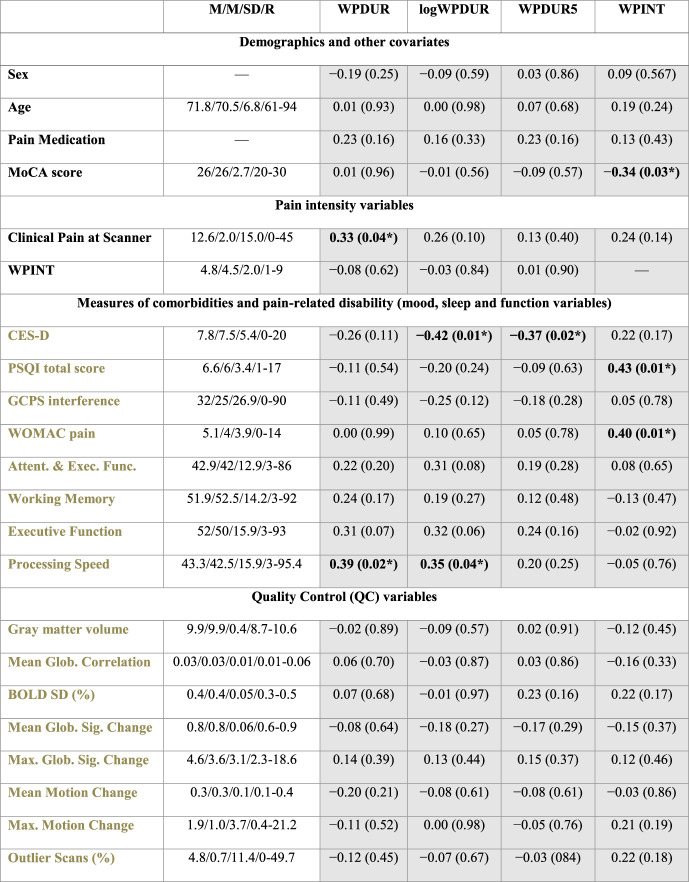

Shaded cells: Pearson correlations (uncorrected *P*-values; *P < 0.05). Missing values were treated using pairwise deletion (the PSQI total score and the NIH toolbox scores had 5 missing data). Except a weak positive correlation with clinical pain at the scanner and with the NIH cognitive toolbox measure of processing speed. Unsurprisingly, WPINT positively correlated with WOMAC pain; as well as with MoCA and PSQI total scores. In addition, logWPDUR and WPDUR5 negatively correlated with CES-D. Gray font: covariates used in post hoc analysis. Clinical pain intensity at the scanner ranges from 0 to 100. WPINT ranges from 0 to 10. Cognitive function variables “attention and executive function,” “working memory,” “executive function,” and “processing speed” are given by the age-corrected individual measure scores “Flanker Inhibitory Control and Attention Test Age 12+,” “List Sorting Working Memory Test Age 7+,” “Dimensional Change Card Sort Test Age 12+,” and “Pattern Comparison Processing Speed Test Age 7+” of the NIH toolbox Cognition Battery, respectively. Gray matter volume is given in 10^5^ mm^3^, and it was calculated from the eroded mask. See the Supplemental Material for details about the measures of comorbidities and pain-related disability. BOLD SD is reported after denoising. The mean global signal and motion changes were calculated after time-point outlier removal. Maximum global signal and motion changes were calculated before outlier removal.

Attent, attention; CES-D, Center for Epidemiologic Studies Depression Scale; Exec, executive; FDR, false-discovery rate; Glob, global; M/M/SD/R, mean/median/standard deviation/range; Med, medication; MoCA, Montreal Cognitive assessment; WPDUR, worst musculoskeletal pain duration; WPINT, worst musculoskeletal pain intensity.

Participants in the control group were between 62 and 85.5 years old, 47% were females, and 20 were right-handed. There were no significant differences in age or MoCA score (independent samples *t* test *P* = 0.6 and *P* = 0.06, respectively), or sex (χ^2^ test *P* = 0.64; Yates corrected), between the pain and control groups.

### 3.1. Connectivity analysis of resting-state magnetic resonance imaging functional connectivity

Table 2 presents, for each approach, the models fitted at each step of the stepwise elimination procedure, the tests performed for each model, and the corresponding *P*-values in case of significance or necessary follow-up analysis. In addition, the effect size of the tested contrasts (as well as the effect size of the covariates) is reported in Table S1 of Supplemental Material S4, available at http://links.lww.com/PR9/A141. The effect sizes of all contrasts of interest were large (Cohen's f^2^ ≥ 0.35), and the effect of clinical pain at the scanner was small to none (f^2^ ≤ 0.15). Details of the significant results under each approach are presented in the sections below.

**Table 2:** *P*-values and estimated false discovery rates (or q-values) of all tests performed in the article.

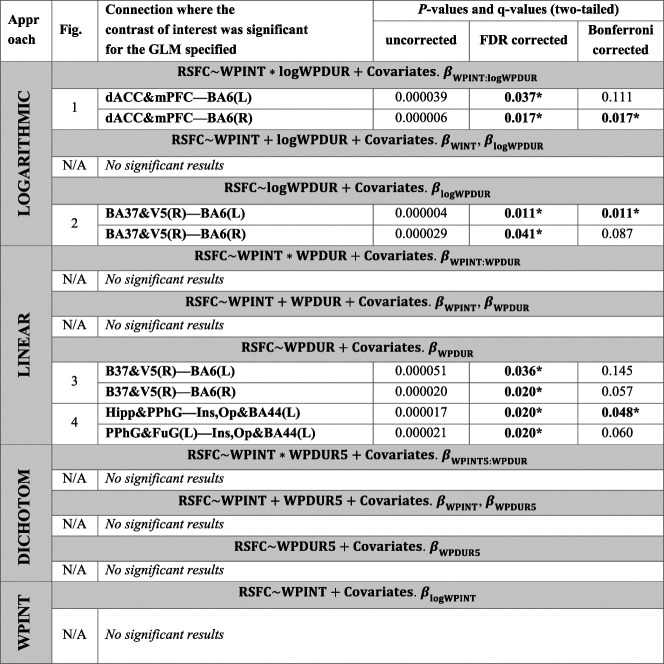

Each test is indicated by the shaded rows with the model and the contrast, separated by a semicolon. The connections and *P*-values of the significant results are shown for each step of the stepwise backward elimination procedure. These order of the steps is shown from top to bottom within each approach, ie, [the connections where β_WPINT:X_ was significant in a model with all terms] → [the connections where β_X_ or β_WPINT_ were significant in the model without WPINT:X term] → [the connections where β_X_ was significant in a model without the WPINT:X and WPINT terms] → [the connections where β_WPINT_ was significant in a model only with the WPINT term (trivially common to all approaches)], being X = {logPDUR, PDUR, PDUR5}. Asterisks (*) denote *P* < 0.05 after correction for multiple comparisons.

BA, Brodmann area; dACC, dorsal anterior cingulate cortex; dmPFC, dorsomedial prefrontal cortex; FDR, false-discovery-rate; FuG, fusiform gyrus; Hipp, hippocampus; Ins, insula; N/A, not available; Op, operculum; PPhG, posterior parahippocampal gyrus; WPDUR, worst musculoskeletal pain duration; WPINT, worst musculoskeletal pain intensity.

### 3.2. Significant associations between pain history variables and resting-state magnetic resonance imaging functional connectivity under the logarithmic approach

**Figure 1.**
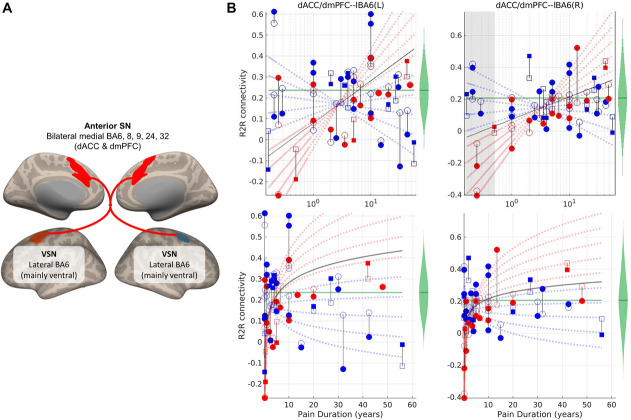
ROI-to-ROI (R2R) connection which resting-state magnetic resonance imaging functional connectivity (RSFC) was significantly associated with WPINT:logWPDUR (*P* < 0.01, FDR corrected; 2-tailed). (A) ROIs and connection (red line: positive interaction). (B) Plots showing the dependency between pain duration and RSFC of the significant connections. Circles and squares correspond to female and male participants with pain, respectively. Filled and empty shapes correspond to observed data and fitted RSFC of participants with pain, respectively. The length of the black lines is equal to the residuals. The green violin plots represent the distribution of RSFC in the group without pain—the average adjusted RSFC within this group is the green horizontal line. In the analysis on the pain group, when WPINT is selected as moderator, the gray lines define the boundaries of the JN regions (WPINT > 6.3 and WPINT > 5.4 for the left and right plots, respectively), where the simple effects of logWPDUR on RSFC are significant. Based on this, we decided to roughly divide the participants into less severe (WPINT ≤ 5) groups (ie, those participants who reported WPINT within at least one of the JN regions after rounding the boundary-defining values to the actual reported values, ie, an integer) and more severe (WPINT > 5)—blue and red shapes thus correspond to participants in the less and more severe groups, respectively. To illustrate this subdivision based on the JN regions, the blue and red lines are the adjusted predicted values for WPINT = {0,1,2,3,4,5} and WPINT = {6,7,8,9,10}, respectively (ie, their slopes are equal to β_logWPDUR_ + β_logWPDUR:WPINT_ WPINT and quantify the simple effects of logWPDUR). Post hoc statistical analysis revealed that the predicted adjusted value of RSFC for a given WPINT was significantly different from the average adjusted RSFC in the control group in the regions where the corresponding line is solid, while not significant where the line is dashed (see Supplementary Material 8 for details, available at http://links.lww.com/PR9/A141). When logWPDUR is selected as moderator, the shaded area define the JN region (WPDUR < ∼6 months) where the simple effects of WPINT on RSFC are significant—the left plot has a null JN region. BA, Brodmann area; dACC, dorsal anterior cingulate cortex; mPFC, medial prefrontal cortex; L, left; R, right; SN, salience network; VSN, visuospatial network; WPDUR, worst musculoskeletal pain duration; WPINT, worst musculoskeletal pain intensity.

Table 2 and Figure 1 report a significant positive WPINT:logWPDUR interaction in 2 connections between an anterior SN ROI in the medial prefrontal cortex with 2 anterior–superior VSN ROIs in the precentral gyrus. This interaction is interpreted as a moderation of one variable (moderator) on the effect of the other (predictor). In moderation analysis, the Johnson–Neyman (JN) region indicates the values of the moderator for which the simple effects of the predictor is significant, as shown in Figure 1B. When selecting pain intensity (WPINT) as a moderator, the JN regions (restricted by the resulting FDR of the interaction analysis, q = 5.5 × 10^−5^) were WPINT > 6.3 and WPINT > 5.4 for the connections to the left and right VSN nodes, respectively. This helped to classify the participants into a *less severe* (WPINT ≤ 5) group, where the effects of pain duration (simple effects of logWPDUR as predictor) on the RSFC were not significant; and a *more severe* (WPINT > 5) where these effects were significant in at least one connection. When choosing pain duration (logWPDUR) as a moderator, the JN regions were logWPDUR∈∅ and logWPDUR < −0.31 for the connections to the left and right VSN nodes, respectively. This helped to explain the effects of pain on the RSFC. Note that the effect of pain intensity (simple effect of WPINT as predictor) was only significant for WPDUR < ∼6 months, ie, for chronic musculoskeletal pains that had a very recent onset. This means that for this subset of participants with pain, RSFC in these anterior SN-VSN connections seems to be negative and significantly lower than in the less severe participants. In addition, the predicted adjusted values of RSFC in these anterior SN-VSN were negative and significantly lower (*P* < FDR q = 5.5 × 10^−5^) than the positive values observed in normal older adults (controls) for WPDUR < 11 to 12 months.

**Figure 2.**
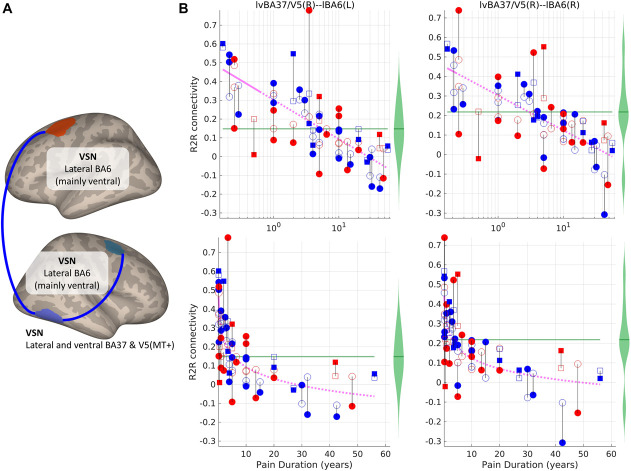
ROI-to-ROI (R2R) connections which resting-state magnetic resonance imaging functional connectivity (RSFC) was significantly associated with logWPDUR (*P* < 0.01, FDR corrected; 2-tailed). (A) ROIs and connections (blue lines: negative effects). (B) Scatter plot showing the dependency between pain duration and RSFC of the significant connections. The magenta line represents the adjusted predicted RSFC. Blue and red shapes correspond to participants with WPINT ≤ 5 and WPINT > 5, respectively. Circles and squares correspond to female and male participants, respectively. Filled and empty shapes correspond to observed data and predicted data, respectively. The length of the black lines is equal to the residuals. The green violin plots represent the distribution of RSFC in the group without pain—the average adjusted RSFC within this group is the green horizontal line. Post hoc statistical analysis revealed that the predicted adjusted value of RSFC was significantly different from the average adjusted RSFC in the control group in the regions where the line is solid, while not significant where the line is dashed (see Supplementary Material 8 for details, available at http://links.lww.com/PR9/A141). BA, Brodmann area; L, left; R, right; VSN, visuospatial network; WPDUR, worst musculoskeletal pain duration; WPINT, worst musculoskeletal pain intensity.

Figure 2 and Table 2 show the significant results in the connections where the backward elimination procedure discarded the interaction term. Independently of pain intensity (WPINT), participants with longer pain duration (logWPDUR) had lower RSFC within the VSN, specifically involving connections between a right occipitotemporal ROI and the same VSN ROIs shown in Figure 1. Figure 2B shows that RSFC between these ROIs within the VSN tended to zero for higher logWPDUR in the pain group; and to higher positive values for lower logWPDUR, even becoming significantly higher (*P* < FDR q = 3.5 × 10^−5^) than the positive average adjusted RSFC in the control group for WPDUR < ∼7 months in the connection to the left VSN node.

### 3.3. Significant associations between pain history variables and resting-state magnetic resonance imaging functional connectivity under the linear approach

**Figure 3.**
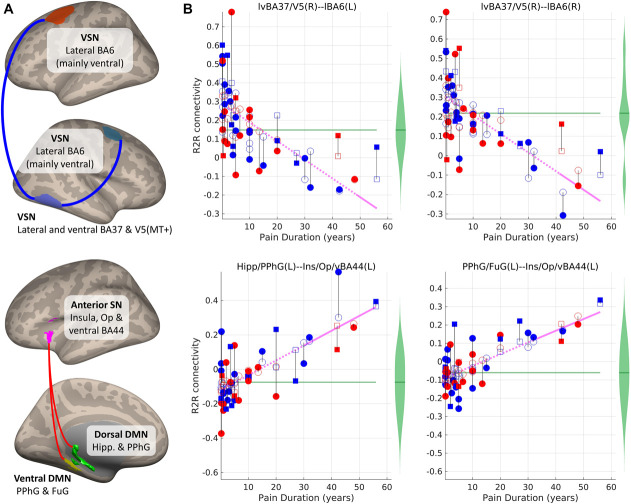
ROI-to-ROI (R2R) connections which resting-state magnetic resonance imaging functional connectivity (RSFC) was significantly associated with WPDUR (*P* < 0.01, FDR corrected; 2-tailed). (A) ROIs and connections (blue and red lines: negative and positive effects). (B) Scatter plot showing the dependency between pain duration and RSFC of the significant connections. The magenta line represents the adjusted predicted RSFC. Blue and red shapes correspond to participants with WPINT ≤ 5 and WPINT > 5, respectively. Circles and squares correspond to female and male participants, respectively. Filled and empty shapes correspond to observed data and predicted data, respectively. The length of the black lines is equal to the residuals. The green violin plots represent the distribution of RSFC in the group without pain—the average adjusted RSFC within this group is the green horizontal line. Post hoc statistical analysis revealed that the predicted adjusted value of RSFC was significantly different from the average adjusted RSFC in the control group in the regions where the line is solid, while not significant where the line is dashed (see Supplementary Material 8 for details, available at http://links.lww.com/PR9/A141). BA, Brodmann area; DMN, default model network; FuG, fusiform gyrus; Hipp, hippocampus; Ins, insula; L, left; Op, opercular; PPhG, posterior parahippocampal gyrus; R, right; SN, salience network; VSN, visuospatial network; WPDUR, worst musculoskeletal pain duration; WPINT, worst musculoskeletal pain intensity.

Figure 3 and Table 2 show the significant results, under the linear approach, in the connections where the backward elimination procedure discarded the WPINT:WPDUR interaction term (all of them). The results in Figures 3A and B are similar to the above-described results in Figure 2 under the logarithmic approach. However, the linear approach detected a different region of significant deviation from the average adjusted RSFC of the control group, ie, WPDUR > ∼40 to 50 years, where RSFC in the pain group was lower than those in the control group in both connections. Furthermore, higher pain durations (WPDUR) were associated with higher RSFC in 2 connections between the anterior SN and the DMNs, as shown in Figures 3C and D. Resting-state MRI functional connectivity tended to zero when WPDUR approached values around 10 to 15 years, and then became positive for higher values of WPDUR, even becoming significantly higher (*P* < FDR q = 7.2 × 10^−5^) than the average in the control group for WPDUR > ∼20 to 25 years, all independently of WPINT.

### 3.4. Post hoc and additional analyses

Supplemental Material S5 (available at http://links.lww.com/PR9/A141) shows that the results presented in Figures 2 and 3 4 were similar to those obtained when using longest pain duration instead of WPDUR. In addition, quantitative diagnostics in Table S2 and Table S3 of Supplemental Material S6, available at http://links.lww.com/PR9/A141, indicate that the assumptions in the linear regressions were not violated and that the significance of the results did not change when removing potential outliers. In addition, the results of a post hoc analysis in Table S4 of the Supplemental Material S7 show that motion and global signal change (QC variables) had small to no effect on the results. Finally, Table S5 of the Supplemental Material S8 reports the correlation between RSFC in the connections where our analysis was significant and sample-descriptive variables of comorbidities and measures of pain-related disability (available at http://links.lww.com/PR9/A141).

## 4. Discussion

There is currently a large body of literature reporting associations between brain morphometric changes and pain duration^3,6,14,19,55,64,71,82^ (although see Refs. 20,^70^ for nonsignificant results). Despite this, to the best of our knowledge, this is the first study to report preliminary significant associations between pain duration and RSFC in a cohort of community-dwelling older adults with musculoskeletal pain with only 3 articles reporting these associations in other age groups and in other pain conditions.^23,38,46^

Musculoskeletal pain seems to have an impact on the anterior SN-VSN RSFC. This impact was only observed on participants with shorter pain durations (specifically around a year or less), for which RSFC seems to have an abnormally negative value in the more severe group; but not in the less severe group—the difference among these 2 severity groups is only observed for pain durations shorter than 6 months. The anterior SN ROI covers part of the dorsal anterior cingulate cortex (dACC) and the dorsomedial prefrontal cortex (dmPFC), both linked to cognitive control functions^16,36,42,51,73,76,81^; and the VSN ROIs predominantly overlap with the premotor lateral Brodmann area 6 (BA6), linked to motor control.^26,32,45^ Our own data in Table S5 of Supplemental Materials S8 (available at http://links.lww.com/PR9/A141) suggest that the RSFC of the affected connections might be positively correlated (although with uncorrected significance) to attention and performance of executive functions. Thus, the reported reduction in RSFC between these ROIs suggest that older adults that had a recent onset of severe chronic musculoskeletal experience alterations in brain mechanism that might lead to some reduction in cognitive control of visuospatial processing or motor actions (with possible detrimental implications on sleep quality; see Table S4, available at http://links.lww.com/PR9/A141). However, Table 1 failed to confirm a relation between pain history and these cognitive functions—we only found an uncorrected correlation between pain duration and processing speed. Future studies should test this hypothesis.

Regardless of pain severity, shorter pain durations (around 7 months or less) seem to be associated with abnormally positive RSFC values in the connections between a VSN ROI surrounding the right occipitotemporal junction (lateral and ventral BA37 and V5/MT+) and the left VSN premotor lateral BA6 ROI. The right lateral BA37 seems to be particularly dedicated to complex visual perception and action observation,^58^ and attention,^7^ whereas the lateral BA6 ROI (covering most of the ventrolateral portion) has also been proposed to have a role in receiving visuospatial information needed for motor control.^45^ This seems to suggest that older adults that had a recent onset of severe chronic musculoskeletal might have altered mechanisms underlying visually guided motor planning or learning. This could be in agreement the positive (but uncorrected) correlation between RSFC and pain-related disability (as measured by Graded Chronic Pain Scale [GCPS] interference; see Table S5, available at http://links.lww.com/PR9/A141). However, we failed to detect a significant correlation between pain history and GCPS that would provide a direct confirmation of this hypothesis. More intriguing, linear modelling suggests that longer pain durations (∼40–50 years or more) seem to be associated to abnormally negative RSFC values in the same VSN connections. This opposite effects in participants with very long pain durations could be related to coping mechanisms developed over the years living with chronic pain. Future neuroimaging studies specifically designed to assess the impact of pain history on physical function would be able to clarify this.

In addition, participants with longer pain durations exhibited abnormally higher RSFC values between the DMN and SN in the left hemisphere, irrespective of pain severity. Note that these 2 networks are normally uncoupled or negatively correlated and that also happened in our control group. The affected connections were between an ROI in the dorsal DMN (partially covering the hippocampus and the posterior parahippocampal gyrus [PPhG]) and an anterior SN ROI (partially covering the insula and opercular and ventral BA44); and between this anterior SN ROI and a ventral DMN ROI (partially covering the PPhG and the fusiform gyrus). This connectivity–pain duration association mirrors that reported in individuals with fibromyalgia (Fig. 2 of Ref. 23). Our findings are consistent with a known role of the hippocampus and insula in chronic pain.^5,12,14,62,66,67,78^ In particular, the left hippocampus is related to memory across the adult lifespan^79^ and pain recall,^37^ whereas the insula encodes the possibility of aversive outcomes.^27^ Thus, greater resting-state hippocampal-insular coupling with longer duration supports the hypothesis that chronic pain is a reverberating process of emotional aversive learning.^4,49^ Future studies are needed to determine if this reflects adaptive mechanisms related to management and coping strategies consolidated at older age, or is a result of impairment in the presence of chronic pain, as previously suggested,^24,43,47,50^ including older populations.^35^

### 4.1. Limitations

This report should be considered as preliminary because it has a small sample size (n = 40), reducing the chance of detecting smaller effect sizes and, more importantly, decreasing the likelihood that statistically significant results actually reflect true effects.^22^ Because this likelihood is inversely proportional to the type I error, lower statistical thresholds would be needed to protect the analysis against possible spurious positive results associated with a smaller sample size, and some of our results with *P*-values close to 0.05 would be considered as marginally significant. This could be circumvented with one-tailed hypotheses (halving *P*-values). For example, the presence of pathological conditions or higher disease burden can be assumed to be associated with lower within network connectivity and higher between network connectivity, as reported for chronic pain within the DMN and between the DMN and SN.^13,24,43,47,50^ This was in fact observed in the aforementioned studies in fibromyalgia, where pain duration positively correlated with DMN-left insular RSFC^23^ and negatively correlated with RSFC within the DMN.^38^ Nevertheless, we expected observable pain duration effects on RSFC with our sample size, given these previous studies detected such associations with smaller sample sizes of 27 and 15.

A second limitation is that the use of the different approaches to model duration–connectivity associations does not fully guarantee capturing their actual nature. This is exposed by the overlap in results (with similar effect sizes and *P*-values) in the connections within the VSN when using the logarithmic and linear approaches, possibly because of a positive skewness in the distribution of pain durations in the sample. Nevertheless, each approach was able to detect a different region of pain durations for which the RSFC of the pain group was significantly different than that of the control group. We deemed justified and necessary to explore all approaches that were already used in the literature to report significant relationships between chronic pain history and neuroimaging. Indeed, although we had overlap, under the linear and logarithmic approaches, we were also able to discover distinct networks or connections that differed in the nature of their pain duration–connectivity relationship, suggesting that different mechanisms could underlie the pain history–related changes in the brain.

Another limitation is that connectivity based on zero-lagged bivariate correlation offers no causal direction, limiting mechanistic interpretation of results. In addition, our results cannot be generalized to younger or middle-aged individuals or cognitively impaired older adults. Finally, our sample is of limited diversity regarding education, socioeconomic status, and race or ethnicity. Future longitudinal studies with larger and more diverse samples, including measures of effective connectivity, would provide further insight into the mechanisms underlying the effects of pain burden from onset to older ages and their relation to comorbidities and behavioral variables.

## 5. Conclusions

Overall, our study suggests that chronic musculoskeletal pain might have a cumulative effect on the brain networks of generally healthy individuals and that these effects are observable at older ages. It also suggests that the strength and direction of these effects might differ depending on when chronic pain onset across the lifespan and how severe it is. Our preliminary results pave the way for future research proposals aiming at investigating how chronic pain may gradually affect executive, motor and cognitive functions, memory, and learning.

## Disclosures

The authors have no conflict of interest to declare.

## Appendix A. Supplemental digital content

Supplemental digital content associated with this article can be found online at http://links.lww.com/PR9/A141.

## Supplementary Material

SUPPLEMENTARY MATERIAL
