# Supplementary material for: Resting-state functional connectivity patterns are associated with worst pain duration in community-dwelling older adults
Source: Pain Rep. 2021 Dec 8;6(4):e978. doi: 10.1097/PR9.0000000000000978 (PMC8660002; doi:10.1097/PR9.0000000000000978)
Supplement: SUPPLEMENTARY MATERIAL [file painreports-6-e978-s001.pdf]

Supplemental Material

S1. Rationale for the inclusion of covariates

We included sex and age given their independent effects on rsfMRI functional connectivity (RSFC) [18,38,47,82,95]. We also accounted for possible current, possibly transient states not necessarily related to the effects of chronic pain burden over the years (or the perception about it) that might confound or mislead the results. Specifically, we accounted for the current use of pain medications, as well as MoCA score, to account for bias in the self-reported pain history variables driven by possible cognitive deterioration. Moreover, it was recently reported that clinical pain intensity at the scanner was associated with resting state DMN-insula RSFC in individuals with fibromyalgia [27]. That study also showed that pain duration was significantly associated with DMN-left insula RSFC in scanner pain-free patients but not in patients with pain at the scanner. This suggests that clinical pain intensity at the scanner could potentially confound the pain duration-RSFC relation examined in the current study, thus we also used it as a covariate.

On the other hand, given that participants already passed the previously described thorough quality control process, QC variables were not added as covariates. Nevertheless, a post-hoc analysis was performed to explore their potential effects on the results (see Table S3 below). Finally, we did not include variables that could be reflecting comorbidities or chronic pain-related traits [i.e., CSE-D, PSQI total score, pain-related physical function (the WOMAC pain subscale and GCPS interference) and NIH toolbox scores; see S2 below] since they might be part of the phenomenon we want to describe, e.g., depression goes alongside pain burden and they could be causally related. A possible correlation between these variables and worst pain duration precludes the use of the

former as adjustment covariates, as it could remove an actual effect of the latter rendering the proposed regression model invalid [73].

S2. Comorbidities and measures of pain-related disability

In order to describe traits and comorbidities that could be associated with the burden of pain, we assessed several measures of mood, sleep and both physical and cognitive function. The Center for Epidemiologic Studies Depression Scale (CES-D) was used to assess, on a 4-point Likert scale, depressive symptoms experienced by participants during the last week. Total scores ranged from 0-60, with higher scores indicating more depressive symptoms [81]. Participants were asked to fill out the Pittsburgh Sleep Quality Index (PSQI)[26] to assess sleep quality. The "PSQI total score" measures the quality and patterns of sleep during the past month. It ranges from 0 to 21, the higher the score the poorer the sleep quality. The Graded chronic pain scale (GCPS) was administered to grade the severity of chronic pain-related interference on physical function over the past 6 months [61]. "GCPS interference" is based on items 4, 5 and 6 with values ranging from 0 to 100. Pain-related disability was also measured using the Western Ontario and McMaster Universities Osteoarthritis Index (WOMAC) [17]. We specifically used the "WOMAC pain" subscale which assesses pain interference with daily activities in the preceding 48 hours. Scores range from 0 to 20, with higher scores indicating greater levels of pain and functional limitations. Finally, measures of cognitive function were also acquired using the NIH Cognition Battery[1] [2,94] as well as the Montreal Cognitive Assesment (MoCA) [62], which assesses global cognitive abilities, including short-term memory, orientation, executive function, language abilities, animal naming,

---

[1] http://www.nihtoolbox.org/WhatAndWhy/Cognition/Cognition%20Battery/Pages/default.aspx

abstraction, attention and clock-drawing. MoCA scores from 0 to 30, with higher scores reflecting higher cognitive abilities.

S3. Effect sizes of the contrasts of interest and the covariates (analysis on the pain group only)

Table S1 shows the effect size of the contrasts tested. According to Cohen's criterion [31], the effect size of the contrasts of interests, i.e., $\beta_{\mathbf{WPINT:logWPDUR}}$, $\beta_{\mathbf{logWPDUR}}$ and $\beta_{\mathbf{WPDUR}}$, on RSFC in the connections shown in Figure 1-4 were large (Cohen's $f^2 \geq 0.35$). On the other hand, clinical pain intensity at the scanner had low or no effect ($f^2 \leq 0.15$) on the RSFC of these connections. Table S1 also reports the effects size of the other covariates.

Table S1. Effect sizes of the covariates

Fig	Connection where the contrast of interest was significant for the GLM specified	Adjusted R^2	Cohen's f^2					
			Contrast tested ($\boldsymbol{\beta}_{\mathbf{X}}$)	Age	Sex	Medic	MoCA	Pain at scan
$\mathbf{RSFC \sim WPINT * logWPDUR + Covariates;}\ \boldsymbol{\beta}_{\mathbf{WPINT:logWPDUR}}$								
**1**	**dACC&mPFC—BA6(L)**	0.47	0.47	0.00	0.05	0.24	0.03	0.11
	**dACC&mPFC—BA6(R)**	0.46	0.46	0.00	0.05	0.00	0.00	0.00
$\mathbf{RSFC \sim logWPDUR + Covariates;}\ \boldsymbol{\beta}_{\mathbf{logWPDUR}}$								
**2**	**BA37&V5(R)—BA6(L)**	0.47	0.45	0.09	0.02	0.00	0.01	0.09
	**BA37&V5(R)—BA6(R)**	0.46	0.39	0.05	0.03	0.00	0.09	0.06
$\mathbf{RSFC \sim WPDUR + Covariates;}\ \boldsymbol{\beta}_{\mathbf{WPDUR}}$								
**3**	**B37&V5(R)—BA6(L)**	0.36	0.36	0.08	0.02	0.00	0.01	0.08
	**B37&V5(R)—BA6(R)**	0.4	0.41	0.05	0.03	0.00	0.09	0.06
**3**	**Hipp&PPhG—Ins,Op&BA44(L)**	0.41	0.40	0.01	0.01	0.15	0.05	0.03
	**PPhG&FuG(L)—Ins,Op&BA44(L)**	0.45	0.45	0.00	0.12	0.16	0.00	0.10

Note. Shaded cells indicate the GLM, determined by the stepwise backward elimination regression, used to reject the null hypothesis that $\boldsymbol{\beta}_{\mathbf{X}} = \mathbf{0}$, where $\mathbf{X}$ is the contrast of interest. The first column shows the Figure where the connection in the second column was reported as significant. Cohen's f2 local effect size index is the proportion of variance explained by the contrast divided by the residual variance. Medic=Current use

of pain medication. dACC=dorsal anterior cingulate cortex. dmPFC=dorsomedial prefrontal cortex. BA=Brodmann area. Hipp=Hippocampus. PPhG=posterior parahippocampal gyrus. FuG=fusiform gyrus. Ins=Insula. Op=operculum.

S4. Using the longest pain duration instead of $\boldsymbol{WPDUR}$

The longest pain duration, i.e., the maximum among the first, second and third worst pain, could also be a measure of the burden of chronic pain. Thus, we evaluated if the results remained after substituting **WPDUR** by the longest pain duration. Probably due to the high correlation between them (r=0.74, $p=5.5\cdot10^{-8}$), the results in Figure 2 partially remained, with the connection to the left VSN surviving significance ($p=9\cdot10^{-6}$ uncorrected; p=0.024, FDR corrected; two-tailed). Also, the results in Figure 3 did not change substantially: $p=5.4\cdot10^{-5}$ and $p=3.5\cdot10^{-5}$ (uncorrected); and p=0.04 and p=0.04 (FDR corrected; two-tailed); for the connections to the left and right VSN nodes respectively. Similarly, for the results in Figure 4, $p=3\cdot10^{-6}$ and $p=6\cdot10^{-5}$ (uncorrected); and p=0.008 and p=0.04 (FDR corrected; two-tailed), for the Hipp&PPhG—Ins,Op&BA44(L) and PPhG&FuG(L)—Ins,Op&BA44(L) connections, respectively.

S5. Diagnostics of the linear regressions

Although the dependent variable, i.e., RSFC, was obtained after a Fisher-transformation of R2R time series correlations to be normally distributed, the independent variable of interest, worst pain duration, was skewed to the left. Therefore, we performed several tests to detect violations of the assumptions of linear regressions in the fits shown in Figure 1-3. We report the Durbin-Watson (DW) test [43] in Table S2. In these regressions, the DW statistics is equal to $2(1-\rho)$, where ρ is the first order neighbor serial correlation of the residuals, and informs about possible departures from the linearity assumption. In our analyses, all DW statistics were close to 2 (i.e., zero serial

correlation) and it was not possible to reject the hypothesis of no serial correlation (all p's $\geq$ 0.34).We also report the Shapiro-Wilk test (or alternatively the Shapiro-Francia test) [86] in Table S1. This is a test of composite normality of the residuals suitable for our sample size. The table shows that we failed to reject the hypothesis of composite normality of the distribution in all linear regressions except two cases. However, their significance did not survive FDR correction for multiple tests across all eight tests.

Table S2. Diagnostics of linear regressions

Fig.	Connection where the contrast of interest was significant for the GLM specified	Pain Group		Control Group	
		Durbin-Watson: Statistics (p-value)	Shapiro-Wilks: Statistics (p-value)	Durbin-Watson: Statistics (p-value)	Shapiro-Wilks: Statistics (p-value)
$\mathbf{RSFC \sim WPINT * logWPDUR + Covariates;}\ \boldsymbol{\beta}_{\mathbf{WPINT:logWPDUR}}$				$\mathbf{RSFC \sim Covariates;}\ \boldsymbol{\beta}_{\mathbf{0}}$	
**1**	**dACC&mPFC—BA6(L)**	2.03 (1.00)	0.98 (0.64)	1.28 (0.08)	0.91 (0.06)
	**dACC&mPFC—BA6(R)**	2.26 (0.47)	**0.93 (0.02)***	1.36 (0.12)	0.93 (0.11)
$\mathbf{RSFC \sim logWPDUR + Covariates;}\ \boldsymbol{\beta}_{\mathbf{logWPDUR}}$				$\mathbf{RSFC \sim Covariates;}\ \boldsymbol{\beta}_{\mathbf{0}}$	
**2**	**BA37&V5(R)—BA6(L)**	1.94 (0.78)	**0.91 (0.01)***	2.32 (0.43)	0.95 (0.33)
	**BA37&V5(R)—BA6(R)**	1.76 (0.40)	0.98 (0.60)	1.54 (0.28)	0.96 (0.54)
$\mathbf{RSFC \sim WPDUR + Covariates;}\ \boldsymbol{\beta}_{\mathbf{WPDUR}}$				$\mathbf{RSFC \sim Covariates;}\ \boldsymbol{\beta}_{\mathbf{0}}$	
**3**	**B37&V5(R)—BA6(L)**	1.99 (0.92)	0.94 (0.06)	1.72 (0.52)	0.95 (0.42)
	**B37&V5(R)—BA6(R)**	2.01 (0.97)	0.95 (0.08)	1.52 (0.26)	0.96 (0.47)
**3**	**Hipp&PPhG—Ins,Op&BA44(L)**	1.73 (0.34)	0.95 (0.07)	1.72 (0.52)	0.95 (0.42)
	**PPhG&FuG(L)—Ins,Op&BA44(L)**	2.03 (0.98)	0.98 (0.78)	1.52 (0.26)	0.96 (0.47)

Note. Diagnostics are reported for each connection. For the pain group, shaded cells indicate the GLM, determined by the stepwise backward elimination regression, used to reject the null hypothesis that $\boldsymbol{\beta}_{\mathbf{X}} = \mathbf{0}$, where $\mathbf{X}$ is the contrast of interest. For the control group, shaded cells indicate that a model including only the covariates was fitted and that we were interested in the intercept (average adjusted RSFC). The first column shows the Figure where the connection in the second column was reported as significant. The third and fifth columns report the Durbin-Watson test of linearity assumption (first order neighbor autocorrelation of the residuals). The fourth and sixth columns report the Shapiro-Wilks test of composite normality (with unspecified mean and variance) of the residuals (for Platykurtic distributions; the Shapiro-Francia test was used Leptikurtic distributions instead). dACC=dorsal anterior cingulate cortex.

dmPFC=dorsomedial prefrontal cortex. BA=Brodmann area. Hipp=Hippocampus. PPhG=posterior parahippocampal gyrus. FuG=fusiform gyrus. Ins=Insula. Op=operculum. *p<0.05 (uncorrected).

Finally, we also reran the linear regressions after removing outliers, i.e., those participants having a Cook's distance higher than 3 times the sample average [32]—outliers based on Cook's distance were no more than 4 for each fit. In all regressions on the pain group, the p-values of the contrasts of interests considerably decreased the adjusted R^2 increased, suggesting that these outliers were not driving the analysis. Figure S1 is a replication of Figures 1-3 but removing the outliers based on Cook's distance.

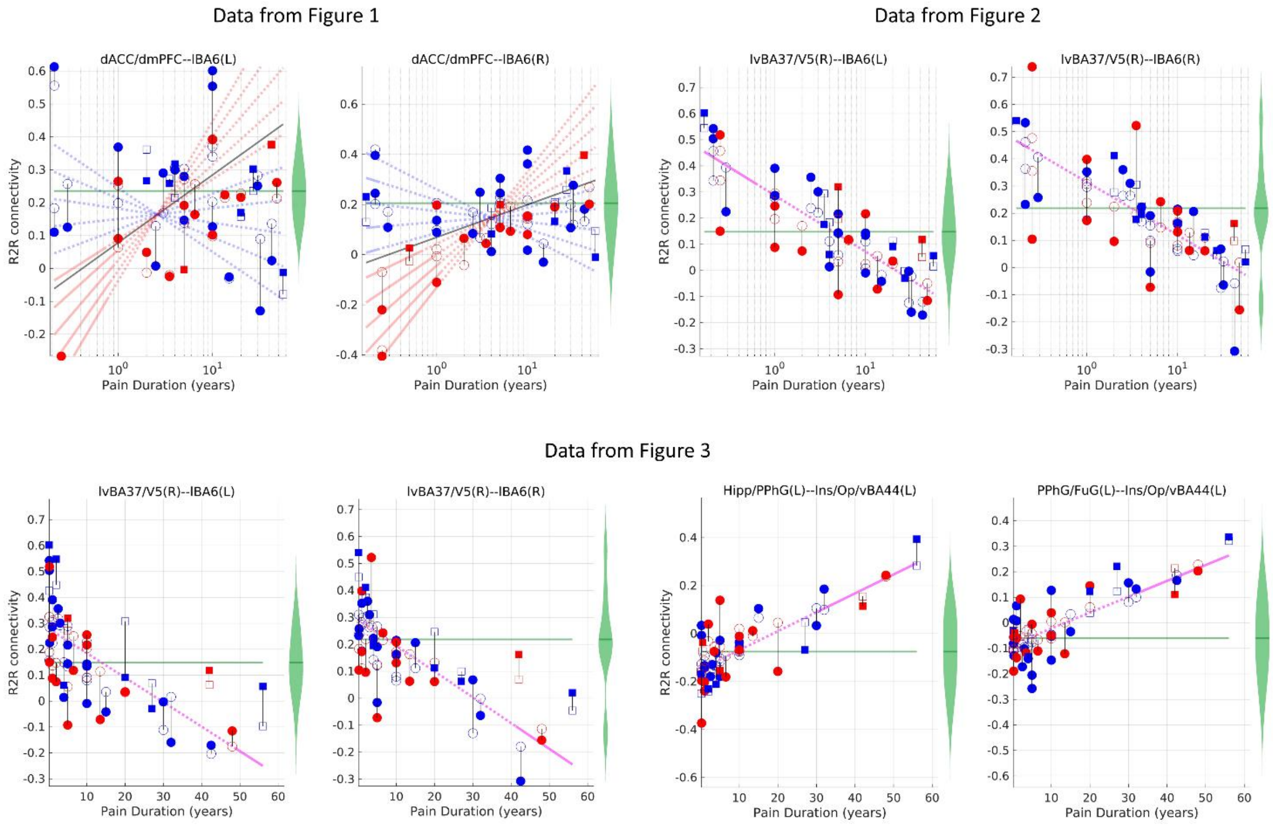

Figure S1. See Figures 1-3 captions.

Similarly, p-values of the contrasts of interests decreased and adjusted R^2 increased when removing the three participants in the pain group tagged as potential outliers based on their number of outlier scans.

Table S3 shows the p-values and adjusted R-squared values before and after removing the all outliers.

Table S3. Effect of the outliers in the linear regressions on the pain group

Fig.	Connection where the contrast of interest was significant for the GLM specified	p-values		Adjusted R^2	
		Before outlier removal	After outlier removal	Before outlier removal	After outlier removal
$\mathbf{RSFC \sim WPINT * logWPDUR + Covariates;\ \boldsymbol{\beta}_{WPINT:logWPDUR}}$					
**1**	**dACC&mPFC—BA6(L)**	$3.9 \cdot 10^{-5}$	$7.7 \cdot 10^{-6}$	0.58	0.67
	**dACC&mPFC—BA6(R)**	$5.9 \cdot 10^{-6}$	$4.0 \cdot 10^{-6}$	0.57	0.66
$\mathbf{RSFC \sim logWPDUR + Covariates;\ \boldsymbol{\beta}_{logWPDUR}}$					
**2**	**BA37&V5(R)—BA6(L)**	$3.9 \cdot 10^{-5}$	$1.1 \cdot 10^{-8}$	0.53	0.76
	**BA37&V5(R)—BA6(R)**	$2.9 \cdot 10^{-5}$	$1.8 \cdot 10^{-6}$	0.48	0.60
$\mathbf{RSFC \sim WPDUR + Covariates;\ \boldsymbol{\beta}_{WPDUR}}$					
**3**	**B37&V5(R)—BA6(L)**	$5.1 \cdot 10^{-5}$	$3.9 \cdot 10^{-5}$	0.46	0.58
	**B37&V5(R)—BA6(R)**	$2.0 \cdot 10^{-5}$	$3.2 \cdot 10^{-7}$	0.49	0.69
**3**	**Hipp&PPhG—Ins,Op&BA44(L)**	$1.7 \cdot 10^{-5}$	$3.8 \cdot 10^{-7}$	0.50	0.70
	**PPhG&FuG(L)—Ins,Op&BA44(L)**	$2.1 \cdot 10^{-5}$	$1.6 \cdot 10^{-5}$	0.53	0.62

Note. Diagnostics are reported for each connection. For the pain group, shaded cells indicate the GLM, determined by the stepwise backward elimination regression, used to reject the null hypothesis that $\boldsymbol{\beta}_{\mathbf{X}} = \mathbf{0}$, where $\mathbf{X}$ is the contrast of interest. dACC=dorsal anterior cingulate cortex. dmPFC=dorsomedial prefrontal cortex. BA=Brodmann area. Hipp=Hippocampus. PPhG=posterior parahippocampal gyrus. FuG=fusiform gyrus. Ins=Insula. Op=operculum.

S6. Post-hoc effect sizes of the QC variables (analysis on the pain group data only)

In an additional post-hoc analysis, we explored possible influence of motion and intensity artifacts on the RSFC of the connections shown in Figures 1-4. To that end, we reran the linear regressions

after adding the QC variables in Table 1 as covariates, one at a time. We found that the effect of each of the QC variables on RSFC was weak (Cohen's $f^2 < 0.15$) in every case except for the GM volume effect on the connection between the left PPhG/FuG node in the ventral DMN and the left insula/opercular/ventral B44 node in the anterior SN, where the effect was moderate (Cohen's $f^2 = 0.26$). In all cases, the p-values of the contrasts of interest did not change substantially, which suggests that QC variables do not significantly explain the variance of RSFC, but rather the contrasts of interest in these connections do. These results are shown in Table S3.

Table S4. Effect sizes of the QC variables

**Fig**	**Connection where the contrast of interest was significant for the GLM specified**	**Cohen's f^2**							
		Gray matter volume	Mean Glob. Correlation	BOLD SD (%)	Mean Glob. Sig. Change	Max. Glob. Sig. Change	Mean Motion Change	Max. Motion Change	Outlier Scans (%)
$\mathbf{RSFC \sim WPINT * logWPDUR + Covariates;\ \boldsymbol{\beta}_{WPINT:logWPDUR}}$									
1	**dACC&mPFC—BA6(L)**	0.05	0.05	0	0.01	0.04	0	0.02	0.05
	**dACC&mPFC—BA6(R)**	0.01	0.04	0.01	0.04	0	0.12	0.03	0
$\mathbf{RSFC \sim logWPDUR + Covariates;\ \boldsymbol{\beta}_{logWPDUR}}$									
2	**BA37&V5(R)—BA6(L)**	0.07	0	0.02	0.06	0.06	0.01	0.04	0.08
	**BA37&V5(R)—BA6(R)**	0.02	0	0.03	0.02	0.03	0.03	0.02	0.04
$\mathbf{RSFC \sim WPDUR + Covariates;\ \boldsymbol{\beta}_{WPDUR}}$									
3	**B37&V5(R)—BA6(L)**	0.03	0.02	0.03	0.12	0.02	0.03	0.06	0.06
	**B37&V5(R)—BA6(R)**	0	0.02	0.01	0.06	0.01	0.06	0.04	0.03
3	**Hipp&PPhG—Ins,Op&BA44(L)**	0	0.03	0	0	0.04	0.04	0.09	0.02
	**PPhG&FuG(L)—Ins,Op&BA44(L)**	0.26	0.15	0.01	0.04	0	0	0	0

Note. Shaded cells indicate the GLM, determined by the stepwise backward elimination regression, used to reject the null hypothesis that $\boldsymbol{\beta}_{\mathbf{X}} = \mathbf{0}$, where $\mathbf{X}$ is the contrast of interest. The first column shows the Figure where the connection in the second column was reported as significant. Cohen's f2 local effect size index

is the proportion of variance explained by the contrast divided by the residual variance. dACC=dorsal anterior cingulate cortex. dmPFC=dorsomedial prefrontal cortex. BA=Brodmann area. Hipp=Hippocampus. PPhG=posterior parahippocampal gyrus. FuG=fusiform gyrus. Ins=Insula. Op=operculum.

S7. Correlation between RSFC and comorbidities and measures of pain-related disability

Table S4 shows the partial Pearson correlation (controlling for the covariates) between the RSFC that was associated with the pain duration variables (see Table 2) and variables measuring possible comorbidities and disabilities, i.e., depressive mood, sleep problems and physical and cognitive function. The RSFC between the node anterior SN node (covering part of the dACC and the mPFC) and the VSN node in the left lateral BA6 correlated with several variables of the NIH cognitive toolbox; while the RSFC between the former node and the right lateral BA6 correlated with PSQI total score. Also, the RSFC between a node in the right BA37/V5 and the right lateral BA6, both within the VSN, correlated with GCPS interference. However, none of these correlations survived correction for multiple comparisons across the tests in the table (p>0.05; FDR corrected).

Table S5. Statistical relation between the RSFC that correlated with the pain duration variables and cognitive, mood traits and physical function.

Variable	$RSFC_1$	$RSFC_2$	$RSFC_3$	$RSFC_4$	$RSFC_5$	$RSFC_6$
**CES-D**	-0.09 (0.60)	-0.27 (0.11)	0.10 (0.56)	0.15 (0.40)	-0.16 (0.37)	-0.21 (0.22)
**PSQI total score**	-0.35 (0.06)	**-0.46 (0.01*)**	0.17 (0.37)	0.17 (0.37)	-0.03 (0.89)	-0.17 (0.37)
**GCPS interference**	-0.15 (0.38)	-0.16 (0.36)	**0.38 (0.02*)**	0.17 (0.34)	-0.25 (0.15)	-0.02 (0.92)
**WOMAC pain**	-0.02 (0.90)	0.12 (0.50)	0.18 (0.31)	0.01 (0.94)	0.06 (0.74)	-0.11 (0.53)
**Attent. & Exec. Func.**	**0.38 (0.04*)**	0.32 (0.10)	0.09 (0.65)	0.15 (0.44)	-0.04 (0.84)	-0.16 (0.42)
**Working Memory**	0.29 (0.12)	0.03 (0.89)	0.02 (0.91)	0.13 (0.48)	0.09 (0.63)	0.13 (0.48)
**Executive Function**	**0.47 (0.01*)**	**0.42 (0.02*)**	0.07 (0.70)	0.08 (0.67)	0.01 (0.96)	-0.08 (0.66)
**Processing Speed**	**0.45 (0.01*)**	0.07 (0.71)	-0.03 (0.88)	-0.02 (0.92)	0.12 (0.55)	0.27 (0.14)

Note. The table shows the partial Pearson correlations (controlling for the covariates). Cognitive function variables "Attention and Executive Function", "Working Memory", "Executive function" and "Processing Speed" are given by the age-corrected individual measure scores "Flanker Inhibitory Control and Attention Test Age 12+", "List Sorting Working Memory Test Age 7+", "Dimensional Change Card Sort Test Age 12+" and "Pattern Comparison Processing Speed Test Age 7+" of the NIH toolbox Cognition Battery, respectively. MoCA=Montreal Cognitive Assesment. CES-D=Center for Epidemiologic Studies Depression Scale. Attent=Attention. Exec=Executive. Func=Function. $RSFC_1$=dACC&mPFC—BA6(L). $RSFC_2$=dACC&mPFC—BA6(R). $RSFC_3$=BA37&V5(R)—BA6(L). $RSFC_4$=BA37&V5(R)—BA6(R). $RSFC_5$=Hipp&PPhG—Ins,Op&BA44(L). $RSFC_6$=PPhG&FuG(L)—Ins,Op&BA44(L). dACC=dorsal anterior cingulate cortex. dmPFC=dorsomedial prefrontal cortex. BA=Brodmann area. Hipp=Hippocampus. PPhG=posterior parahippocampal gyrus. FuG=fusiform gyrus. Ins=Insula. Op=operculum. *p<0.05 (uncorrected).

S8. Determination of the pain durations for which RSFC was significantly difference between pain and control groups.

Written in Wilkinson notation, the models $\mathbf{RSFC \sim WPINT * X + Covariates}$ ($\mathbf{X}$ a pain duration variable) and $\mathbf{RSFC \sim Covariates}$ were fitted to the RSFC data in the pain and control group, respectively. Thus, the two independent samples t-test of significant group difference in the fitted RSFC values adjusted for the covariates is given by:

$$\mathbf{T(df,q)} = \frac{\boldsymbol{\beta_0 - \beta_0^{(c)} + \beta_X X + \beta_{WPINT} WPINT + \beta_{WPINT:X} XWPINT}}{\sqrt{\begin{array}{c}\boldsymbol{\sigma^2(\beta_0) + \sigma^2\left(\beta_0^{(c)}\right) + \sigma^2(\beta_X)X^2 + +\sigma^2(\beta_{WPINT})WPINT^2 + \sigma^2(\beta_{WPINT:X})X^2WPINT^2} + \cdots \\ \cdots + \boldsymbol{2cov(\beta_0, \beta_X)X + 2cov(\beta_0, \beta_{WPINT})WPINT + 2cov(\beta_0, \beta_{WPINT:X})XWPINT} + \cdots \\ \cdots + \boldsymbol{2cov(\beta_X, \beta_{WPINT})XWPINT + 2cov(\beta_X, \beta_{WPINT:X})X^2WPINT + 2cov(\beta_{WPINT}, \beta_{WPINT:X})XWPINT^2}\end{array}}}$$

Where $\boldsymbol{\beta_0}$ and $\boldsymbol{\beta_0^{(c)}}$ are the estimated intercepts of the models for the pain and control groups; $\boldsymbol{\beta_X}$, $\boldsymbol{\beta_{WPINT}}$ and $\boldsymbol{\beta_{WPINT:X}}$ are the estimated coefficients of the effects of $\mathbf{X}$, $\mathbf{WPINT}$ and the interaction $\mathbf{WPINT:X}$, respectively; $\boldsymbol{\sigma^2(\beta_A)}$ is the estimated variance of the coefficient of term $\mathbf{A}$; and $\mathbf{cov}(\boldsymbol{\beta_A, \beta_B})$ is the covariance between the coefficient of terms $\mathbf{A}$ and $\mathbf{B}$ (note that $\boldsymbol{\beta_0^{(c)}}$ is independent from the coefficients estimated in pain group).

For a given fixed value of $\mathbf{WPINT}$ (when the interaction model was significant) or for $\mathbf{WPINT} = \mathbf{0}$ (when neither the interaction nor the $\mathbf{WPINT}$ term for significant), we calculated the roots of the quadratic function that resulted from squaring the equation above. This yielded the regions in the domain of $\mathbf{X}$ where the group difference in fitted RSFC values were significant, shown in Figure 1-3 with solid lines (versus dashed for the region of no significance). The value of $\mathbf{T}(\mathbf{df}, \mathbf{q})$ was calculated for the estimated degrees of freedom and the post-hoc FDR q-value threshold established by fitting the models to the pain group. To estimate the degrees of freedom, we explored both assumptions of equal ($\mathbf{df} = \mathbf{n_p} + \mathbf{n_C} - \mathbf{2}$) and unequal (Welch's formula) variance—since df depends on the coefficients for the latter assumption, we calculated the average across all values of $\mathbf{X}$. Results using both assumptions were almost identical.
